# Coping Mechanisms of Previously Diagnosed and New HIV-Discordant, Heterosexual Couples Enrolled in a Pilot HIV Self-Testing Intervention Trial in Central Uganda

**DOI:** 10.3389/frph.2021.700850

**Published:** 2021-08-24

**Authors:** Joseph K. B. Matovu, Rose Kisa, Angela M. Malek, Caroline Vrana-Diaz, Semei Christopher Mukama, William Musoke, Jeffrey E. Korte, Rhoda K. Wanyenze

**Affiliations:** ^1^Department of Disease Control and Environmental Health, School of Public Health, College of Health Sciences, Kampala, Uganda; ^2^Department of Community and Public Health, Busitema University Faculty of Health Sciences, Mbale, Uganda; ^3^Department of Public Health Sciences, College of Medicine, Medical University of South Carolina, Charleston, SC, United States; ^4^Research Department, Mildmay Uganda, Kampala, Uganda

**Keywords:** coping mechanisms, HIV self-testing, HIV-discordant, couples, Uganda

## Abstract

**Introduction:** Learning that a couple has HIV-discordant results can create tensions in the relationship including separation. We explored the coping mechanisms of HIV-discordant, heterosexual couples enrolled in an HIV self-testing (HIVST) intervention trial in Central Uganda.

**Materials and Methods:** This qualitative study was nested within a pilot HIVST intervention trial targeting pregnant women and their male partners in central Uganda. In-depth interviews were conducted with 18 individuals from 13 HIV-discordant couples between July and September 2018; 18 months after the end of the main trial. Data were collected on the couples' initial reactions after learning about their HIV-discordant status, mechanisms adopted by couples to cope with HIV-discordance, and suggestions on how couples in similar situations can be supported. Interviews were transcribed verbatim and analysed manually following a thematic framework approach.

**Findings:** Of the 13 HIV-discordant couples, the female partner was HIV-positive (M–F+) in seven, while the male partner was HIV-positive (F–M+) in six. The mean (±SD) age of the participants was 32.6 (±6.4) years and participants had stayed together for an average of 5.5 (±3.6) years. Fourteen participants from nine couples already knew about their HIV-discordant status by the time they participated in the HIVST trial. After learning about their HIV-discordant status, most individuals (15) thought of abandoning their relationship; three (3) thought of committing suicide. To cope with HIV-discordance, some couples reported that they sought professional counselling support from healthcare providers, and this was particularly true for couples that were already aware of their HIV-discordant status by the time they participated in the HIVST trial. However, new couples that learnt about their HIV-discordant status after participating in the trial reported that they sought psycho-social support from friends or relatives. In the majority of cases, couples reported that they reduced the frequency of sex or abstained from sex. Some couples temporarily separated from their partners, while a few others resorted to using condoms to reduce HIV infection risk.

**Conclusion:** Couples used a variety of approaches to cope with HIV-discordance. Study findings underscore the importance of ongoing professional counselling and psycho-social support in helping couples to cope with HIV-discordance.

## Introduction

Advances in HIV prevention, care, and treatment have improved the ability for HIV-discordant couples (i.e., couples in which one of the partners has HIV) to have a meaningful sexual life and achieve safer conception without the risk of the uninfected partner acquiring HIV from their HIV-positive partner (1–3). A prospective, observational PARTNER (Partners of People on ART-A New Evaluation of the Risks) study among 888 heterosexual HIV-discordant couples in which the HIV-positive partner was using suppressive antiretroviral therapy (ART) found no documented cases of within-couple HIV transmission over 1,238 couple-years of follow-up even though couples engaged in condomless sex for a median of 37 times per year (4). In the Partners Demonstration Project, an open-label study of the integrated delivery of Pre-Exposure Prophylaxis (PrEP) and ART among mutually-disclosed high risk HIV-discordant couples in Kenya and Uganda, Heffron et al. (5) reported no HIV seroconversions among 1,013 HIV-discordant couples that planned to have a baby due to the fact that 82.9% of the couples reported using PrEP or ART in the 6 months preceding the pregnancy. These findings reaffirm previous results from the HPTN (HIV Prevention Trials Network) 052 study which reported a 96% reduction in the risk of HIV transmission in HIV-positive individuals randomised to receive early ART compared to those randomised to delayed treatment (6).

However, despite these advances, living in an HIV-discordant relationship can have its own challenges (7–9). These challenges range from confusion and disbelief around HIV-discordance itself (10–12) to anger, confusion (as to whether they should terminate the relationship or decide to stay in the marriage), and shock (resulting from the perception that their trusted marital partners were having sex with other sexual partners, and that they most likely got infected with HIV from such relationships) particularly among couples learning about their HIV-discordance for the first time (9, 13). Given this state of confusion, shock and fear of the unknown, it is imperative to understand how couples in such relationships cope with the knowledge that one of the partners is HIV-positive while the other one is not.

While previous studies have explored the topic of coping mechanisms among HIV-discordant couples and we have a clear understanding of the challenges that such couples grapple with, these studies were conducted among couples that had gone through professional pre-test and post-test counselling. With the emergence of HIV self-testing (HIVST), a process through which an individual can perform the HIV test and learn about their HIV results without the support of a professional provider, it is now possible that couples can learn about their HIV-discordant status after HIVST, without the presence of a professional healthcare provider. Studies suggest that HIVST is slowly becoming the game changer in improving couples' HIV testing uptake particularly in sub-Saharan Africa where couples' HIV testing remains stubbornly low over the years (14, 15). Thus, as HIVST becomes increasingly available, more and more couples will test for HIV together with their partners using this alternative HIV testing strategy. However, if the couple learns about their HIV-discordance without the help of a professional healthcare provider, it is more likely that they may fail to appreciate their HIV-discordant status or the results may trigger cases of marital disruptions and suicidal ideations (16, 17). In one study that was conducted among self-tested, HIV-discordant couples, there were no cases of adverse reactions reported after self-testing but a few couples were reported to have separated after learning about their HIV-discordant status (13). Thus, it is likely that HIV-discordant couples that self-test for HIV will experience more challenges in coping with their HIV-discordant status compared to other types of couples that have received professional pre- and post-test counselling. At the moment, few studies have explored how couples cope with HIV-discordance in the context of HIVST. The purpose of this study was to explore the coping mechanisms of HIV-discordant couples after HIVST to inform the design of interventions to support HIV-discordant couples who self-test for HIV.

## Materials and Methods

### Study Site

This study was conducted at three health facilities (Entebbe Hospital, Mpigi Health Centre IV and Nakaseke Hospital) in Central Uganda as part of a HIVST intervention trial to improve HIV testing uptake and linkage to care among male partners of pregnant women receiving antenatal care services at the above-mentioned health facilities. The methodology for the HIVST trial has been described previously (14, 18). In brief, the trial enrolled 1,514 pregnant women and randomised them into the intervention (777) and control arm (737). Intervention arm participants received two HIV self-test kits (one for the woman and the other for her male partner) to use in self-testing for HIV; in the control arm, women were asked to encourage their male partners to go to the nearest health facility to test for HIV.

### Study Design and Population

This was a qualitative study nested within the above-mentioned HIVST intervention trial. Study participants were HIV-discordant couples identified from the HIVST trial. Participants were contacted for this study 18 months after they last participated in the HIVST trial. This timing was a result of the phased nature of the primary HIVST intervention trial and thus coincided with the last phase of the main trial.

### Participants' Selection

There were seventy-five HIV-discordant couples that were enrolled into the HIVST intervention trial. Of these, 13 couples (i.e., 26 individuals) were selected to participate in this study. The decision to select 13 couples was based on logistic considerations, given that the study was conducted 18 months after the end of the main trial. We thought that it would be difficult to trace all the 75 couples after a long duration since the study ended. So, we decided to select couples from across the three sites while ensuring a fair representation of couples in which the male partner was HIV-positive and those in which the female partner was HIV-positive. Indeed, in line with our fears, of the 26 individuals that we expected to enrol from 13 couples, only 18 individuals were successfully contacted and invited to come for interview while 8 participants could not be reached. Individuals that were successfully contacted were either visited in their homes (if they indicated inability to come to the study site) or invited to come to the study site for a face-to-face in-depth interview.

### Data Collection Methods

Data collection took place between July and September 2018 (18 months after the end of the HIVST trial) using an in-depth interview guide developed by JKBM and RK. Interviews took place after obtaining written informed consent from the participants. All interviews, including those with paired participants (i.e., members of the same couple) were interviewed separately. The term “*coping mechanism*” was defined as the various ways through which couples in an HIV-discordant relationship tried to understand, appreciate, and live together as a couple despite the fact that they had different HIV test results. Interview questions ranged from questions on how the couple reacted upon learning that one of the partners had HIV (e.g., *What went through your mind on the first day that you learnt of your HIV-discordant status?*); questions on coping with HIV-discordant results (e.g., *Did you receive any form of support—e.g., counselling—from anyone or any organisation during the period after learning about your HIV-discordant status? If you received any support, what form of support did you receive? How did it help you to continue to live with your partner? If not, how were you able to cope with the knowledge of your partner's HIV status (which was different from yours) without any support?*); questions about changes that have happened since the couple learnt about their HIV-discordant status (e.g., *Having one partner living with HIV when the other one does not have it can be challenging to your sexual intimacy. Please tell me about any changes that you have introduced in your sexual intimacy as a result of knowing that you have different HIV status. How have these changes helped to keep your sexual relationship intact?*) and general questions on HIV-discordance (e.g., *What advice or suggestion would you give to a couple that experiences HIV-discordance?*). Participants were asked about the duration of their sexual relationship (i.e., for how long they had been living together) and whether or not they learnt about their HIV-discordance for the first time after participating in the HIVST trial. Six in-depth interviews were conducted at each health facility by a team of three trained Social Scientists with experience in the conduct of qualitative interviews. All members of the study team had a minimum of a Bachelor's degree. All interviews were audio-recorded with permission from the participants and lasted about 1 h 30 min, on average. Interviews were transcribed verbatim within 1–2 days of data collection.

### Data Analysis

Data analysis was conducted using a theoretical framework approach. Data were analysed deductively, using a realist/essentialist framework, at a semantic level, following the six steps suggested by Braun and Clarke (19). These steps include: 1. Becoming familiar with the data; 2. Generating initial codes; 3. Searching for themes; 4. Reviewing themes; 5. Defining themes and 6. Writing the research report. As noted by Braun and Clarke (19), we did not follow these steps in a linear fashion; instead, we constantly moved between steps, back and forth, during the process of analysing the data. This process enabled us to become familiar with the data. Data analysis was done with the four *a priori* themes in mind (*initial reactions after learning about HIV-discordance; coping mechanisms; changes in sexual intimacy, and advice to couples in a similar situation*) while noting any emerging themes along the way. Initially, JKBM and RK identified six transcripts (i.e., two from each site) and read them to identify key issues that pertained to each of the four *a priori* themes. After this initial coding, a list of codes was generated that was applied to the rest of the transcripts to complete the coding process. We then created a matrix (in MS Excel) of issues emerging from the data, arranged by the four *a priori* themes. We identified, from each transcript, quotations that pertained to the issues emerging from the data and entered them into the Excel sheet. We then read through the quotations to identify any patterns emerging from the data. As we read through the quotations, we realised that quotations initially placed under the theme, “*changes in sexual behaviour*” were similar to those that we had placed under “*coping mechanisms*.” We therefore merged the two themes into one and retained “coping mechanism” as the primary theme. Besides, we realised that there were quotations that could not fit well within the remaining three *a priori* themes, including the misconception that HIV transmission is related to one's blood group and the fears from HIV-negative partners that one day their HIV-positive partners will infect them with HIV. A new theme was thus created and named, “*current thoughts and fears about HIV-discordance*.” We then re-arranged the quotations (according to which issue they supported most) while ensuring that those that addressed a similar or related issue were put close to each other. This enabled us to create sub-themes under each of the five themes. Finally, we identified quotations that supported each sub-theme, and used these in reporting the findings. We followed the consolidated criteria for reporting qualitative studies (COREQ) in reporting the findings (20).

### Ethical Considerations

The study protocol was approved by Makerere University School of Public Health Higher Degrees, Research and Ethics Committee (Protocol#: 392) and cleared by the Uganda National Council for Science and Technology (HS 2022); in accordance with the Helsinki Declaration of 1975, as revised in 2008. Participants provided written informed consent prior to their participation in the study.

## Results

Eighteen individuals out of the 26 individuals in 13 HIV-discordant couples were interviewed about how they coped with their HIV-discordant status since they last self-tested for HIV. Of the 18 participants, 10 were in paired relationships (i.e., both male and female partners were interviewed, though separately) while eight were interviewed as the only partner from the couple relationship. We were not able to interview the eight partners of those who were interviewed alone because they either refused to participate (2) or were not available at the time of interview (6). However, although we did not interview all the 26 individuals as expected, we were able to interview at least one partner from each couple. Of the 13 HIV-discordant couples, seven had the male partner HIV-negative (M–F+) while six had the female partner HIV-negative (F–M+).

The mean (±SD) age for the participants was 32.6 (±6.4) years and participants had stayed together for an average of 5.5 (±3.6) years. Most of the individuals (14) were already aware of their HIV sero-discordance by the time they were enrolled in the HIVST trial. These individuals were drawn from nine couples; five in which both partners were interviewed and four in which only one partner (either the male or female partner) was interviewed (see items 1.1–5.2, Table 1). Half of the participants had secondary education while the other half had primary education. All individuals discovered their HIV sero-discordant status after they had begun cohabitating with their husband/wife. That is, there was no reported attempt by the couples to test for HIV prior to relationship formation even though most of the HIV-positive partners acknowledged that they were already aware of their HIV-positive status at the time of relationship formation.

**Table 1:** Characteristics of the eighteen individuals interviewed for this study.

**#**	**Partner type**	**Age**	**Education**	**Marital duration**	**Already knew HIV-discordance status by the time of the trial (Y/N)**
1.1	HIV-negative male partner	32	Secondary	6 years	Yes
1.2	HIV-positive female partner	22	Primary	6 years	Yes
2.1	HIV-negative male partner	45	Primary	14 years	Yes
2.2	HIV-positive female partner	32	Secondary	14 years	Yes
3.1	HIV-negative male partner	34	Secondary	4.5 years	Yes
3.2	HIV-positive female partner	25	Secondary	4.5 years	Yes
4.1	HIV-negative male partner	38	Secondary	1.5 years	Yes
4.2	HIV-positive female partner	28	Primary	1.5 years	Yes
5.1	HIV-negative male partner	42	Primary	4 years	Yes
5.2	HIV-positive female partner	36	Secondary	4 years	Yes
6	HIV-negative male partner	40	Primary	3 years	No
7	HIV-negative female partner	28	Primary	8 years	No
8	HIV-negative female partner	30	Secondary	4 years	Yes
9	HIV-negative female partner	35	Primary	7 years	No
10	HIV-positive male partner	33	Secondary	7 years	Yes
11	HIV-positive male partner	24	Secondary	2 years	No
12	HIV-positive male partner	37	Primary	5 years	Yes
13	HIV-positive female partner	36	Primary	4 years	Yes

Only four individuals, representing four couples (i.e., one couple in which the male partner was HIV-negative and three couples in which the female partner was HIV-negative) learnt about their HIV sero-discordant status after self-testing with their partner. The rest of the couples already knew their HIV-discordant status by the time of participating in the HIVST trial. However, members of the study team did not know whether or not these couples were HIV-discordant at the time of giving them HIV self-test kits; this only became known during the follow-up interviews. Since most of the couples were already aware of their HIV-discordant status by the time of enrolment into the HIVST trial, we explored their coping histories, going back to the time (before the HIVST trial) when they first learnt about their HIV-discordant status. Thus, the results presented in this paper are a mixture of coping histories from most of the couples that were already aware of their HIV-discordant status, as well as coping mechanisms adopted by the four couples that came to know about their HIV-discordant status when they enrolled into the HIVST trial. Study findings have been grouped into four themes: (a) *initial reactions when members of the couple learnt for the first time that they were HIV-discordant*; (b) *how couples coped with their HIV-discordant status and managed to live together until the time of interview*; (c) *current thoughts and fears about HIV-discordance* and (d) *advice to couples in other HIV-discordant situations*.

### Couples' Initial Reactions After Learning About Their HIV-Discordant Status

Our findings suggest that when couples learnt of their HIV-discordant status for the first time, two things happened, depending on which partner was HIV-positive: (i) *HIV-positive partners feared that their HIV-negative partners might abandon the relationship while HIV-negative partners thought of separating from their HIV-positive partners*; and (ii) *some participants developed suicidal ideations but these cleared after they were counselled*, as described in detail below.

#### Initial Thoughts About Abandoning the Relationship

In 15 of the 18 participants interviewed, the initial reaction after learning about their HIV-discordant status was separation from their partner. HIV-negative partners thought of abandoning the relationship partly because of frustration that their partners were HIV-positive and partly because of fear of contracting HIV from their HIV-positive partners. An HIV-negative woman who learnt about their HIV-discordant status when she enrolled into the HIVST trial narrated how she got “*a big challenge*” when she learnt that her husband was HIV-positive, and “*felt stressed*” to the extent that she thought of abandoning the relationship, as the following quotation illustrates:

*I thought of walking away and abandoning him, I thought of separating from him, I even didn't want to have a glance at him at that time but I cooled down, such thoughts would come and I felt so stressed, I was pregnant and felt stressed. But I decided to calm down by myself, the health workers had tried to counsel me that this was possible, I could stay like that and look after my children as he also goes his own ways. Generally, I got a very big challenge and if it was not the fact that I was strong, I would not have tolerated it at all or would have packed my bags and separated from him* (**HIV-negative woman**).

HIV-positive men thought that their HIV-negative female partners would abandon the relationship for fear of contracting HIV from them while HIV-positive women thought that their HIV-negative male partners would chase them out of the home. An HIV-positive male partner who first learnt of their HIV-discordant status during the HIVST trial said that his HIV-negative female partner threatened to abandon the relationship for “*most of the time*”: ‘*We used to discuss issues together but we stopped doing that [when she learnt of my HIV-positive status]. We could not discuss anymore … She was constantly quarrelling. She kept on saying that “I am leaving, I am leaving.” She wanted to leave most of the time*. An HIV-positive woman thought that her husband would “*over-react*” after learning about her HIV-positive status and possibly throw her out of the home. However, as she attests toward the end of the quotation, her husband “*did not react like I had expected*”, something that surprised her:

*What came to my mind, at heart I was so scared and worried and had this feeling that since he had found out that I am HIV-positive while he is HIV-negative, he might over-react and in the end we separate but all in all, I was also relieved after knowing the truth about his status and became strong and ready to go by whichever decision he would make. To my surprise, he did not react like I had expected because he chose to stay with me and support me in my situation* **(HIV-positive woman)**

Although the issue of separation emerged as the initial reaction after the couple learnt about their HIV-discordant status, in the majority of couples, the HIV-negative partner who wanted to abandon the relationship eventually decided to stay with his/her HIV-positive partner. Most HIV-negative partners cited fear of getting into a new relationship with another partner who might as well be HIV-positive. In other instances, the HIV-negative partner actually left the relationship but this was temporary, and they eventually returned to the relationship, as indicated in the quotations below.

…* I thought about leaving [abandoning the marriage] but again I thought that even if I leave this man, I will not stay single forever. It is possible to leave this one whose status I know of and … I land on one who is positive but [who] is not willing to take a test. I will still end up getting infected. This gave me courage to stay with my partner whose status I know and he also understands the fact that he is positive but his partner is negative. In this case he will know how to handle me and I will also know how to handle him in this situation* **(HIV-negative woman)**

*She is insisting to remain my wife. She claims that the children would like to spend most of their time with their father. We had separated in October last year up to around May this year. She came and forced me to have sex with her but I used a condom. I have been having sex with her but I feel uncomfortable. So that day when we went and tested together, she was negative. Then the health worker counselled us and left the decision to be made between us* **(HIV-positive man)**

#### Suicidal Ideations

Two HIV-positive men and one HIV-positive woman indicated that they initially thought of committing suicide after finding out that they were HIV-positive and their partners were HIV-negative, but also due to the fact that their HIV-negative partners were threatening to leave them. An HIV-positive man narrated how he felt that he was no longer “worth living” since his partner who would have consoled him was threatening to leave him. However, when these individuals received psycho-social support from their relatives as well as counselling from the health workers, they managed to drop the idea of committing suicide and decided to continue with life, as the quotation below indicates:

*I had decided to commit suicide. I got that thought so often because of three reasons, first was because I had found myself infected with HIV, second was I was no longer working and thirdly, my wife who would have counselled me was continuously threatening to abandon me. Whenever I thought of all these, I felt like I was not worth living but I dropped the decision because of the thorough [the support hat] I received from my aunt and my parents and also the fact that my wife eventually decided to stay with me in our relationship and we look after our children* **(HIV-positive man)**

### How Couples Coped With Their HIV-Discordant Status

Individuals in HIV-discordant relationships coped with their HIV-discordant status in several ways. Some couples reduced the frequency of sex or abstained from sex, some couples insisted on condom use to reduce the risk of HIV infection to the HIV-negative partner, and some other couples sought counselling and/or psychosocial support from health workers, relatives, or friends, as indicated in the following sub-sections.

#### Reduced Sexual Frequency and/or Sexual Abstinence

Most HIV-negative participants with HIV-positive partners narrated how learning about their HIV-discordant status resulted in reduced sexual frequency or sexual abstinence due to the low sexual desire at the time. HIV-negative partners reported that they took months without having sex with their HIV-positive partners. HIV-positive partners talked of “dodging” their HIV-negative partners because of the fear of infecting them with HIV.

*After realising that I was HIV-positive and he was negative, I started dodging him. He asked me, did you get another man? I told him that no, it is not the case, it is just that I am not in the mood. So basically I put up an environment which was not giving him a chance to have sex with me... In my heart it was haunting me, I kept thinking to myself that “I am going to infect him” [She says it with a tone that indicates regret]. That happened to me and I was feeling guilty in my heart and I kept saying to myself that I am going to kill an innocent boy* **(HIV-positive woman)**

An HIV-negative woman who had always suspected her male partner to be engaged in an extramarital affair eventually found out that her male partner was HIV-positive when he accepted to use the HIV self-test kit. This was the first time that this woman learned about their HIV-discordant status. In the discussion with her HIV-positive partner that ensued, she came to learn that her male partner had actually been on HIV treatment for 2 years although he had never disclosed his HIV-positive status to her. This angered her so much that she decided to abandon sex for “*one year and some months*”; and whenever her partner asked her about sex, she would ask him to “*first leave me alone and give me some space… [and that I would] inform him at the right time when we would engage in sex*.” This woman's case was not an isolated one given that several other HIV-positive individuals did not disclose their HIV-discordant status to their new partners at relationship formation. HIV-negative women in particular only came to learn about their male partners' HIV-positive status after they insisted on testing with them at the nearest health facility during antenatal care visits.

#### Condom use to Reduce HIV Infection Risk

Some couples opted to use condoms to reduce the risk of HIV infection. This was particularly the case among HIV-negative women who insisted that if any sex had to take place, they should use a condom to protect themselves from the risk of HIV infection.

*My attitude changed a bit because he is not the person I can completely open up to currently. I frankly tell him that if he would like to be with me [i.e., have sex with me], he must use a condom and if he does not want, then he is free to go to his other partner. So those are some of the changes that emerged* **(HIV-negative woman)** *I spent months and months and months and months and months [without having sex with him] … It did not come to a year but it was about half a year until we sat and had a discussion about it. He told me … I don't know what will happen if we don't have sex yet we are married. I told him that you have to wear a condom and he said that I agree I will use it. Then I told him that it is fine then. Why I did that is because I was protecting myself but I can't really explain why I got that problem just as you can face a challenge that you can't explain. However, he told me that he was going to give me time until I make up my mind* **(HIV-negative woman)**

However, most of the HIV-negative men did not insist on condom use; and, in some cases, refused to use them even when their HIV-positive female partners insisted on their use. An HIV-negative man with an HIV-positive female partner indicated that knowing that his female partner was HIV-positive did not result in any change in the way he had sex with her. It is important to note that by the time of learning about their HIV-discordant status, this man had already known that his wife was HIV-positive and presumed that he was equally HIV-positive since he had not yet tested for HIV. However, although he was surprised to find out that he was indeed HIV-negative, he still refused to use condoms, arguing that it wasn't necessary since his partner is already receiving HIV treatment.

*I failed [to use a condom] because you may find when you love your wife and she has been taking her drugs very well, I find it hard to start telling her that we start using condoms. Because she may suspect that you want to isolate yourself from her. So when you think that you are trying to protect yourself, this may make her worried even more. She may think that you have started to distance yourself from her. So we are still depending on the fact that she is taking her drugs but I have no guarantee that I will not get infected* **(HIV-negative man)**

Some HIV-positive men reported that consistent condom use was not always possible and sometimes they would be tempted to have unprotected sex. In four couples, men indicated that they did not use condoms to reduce the risk of HIV infection, despite encouragement from their partners. Some men reported that they did not have a single condom in the house (as proof that they were not inclined to use them) while others indicated that they actually had condoms in the house (which they received from the health facilities) but had never used them.

*I have never even suggested that we use condoms. Everything remained the same… Doctor, even the condoms we were given are still available in the house, we have never used them* **(HIV-negative man)**

HIV-positive female partners mentioned that although they had tried to indicate to their male partners that unprotected sex would expose them to HIV infection, some of their HIV-negative male partners totally refused to use condoms, as illustrated in the quotation below:

*There is something that surprises me about that man; he is a very “brave” man. I often tell him that we should use a condom and he says that what for? Then I tell him that I don't want you to get infected especially if you are infected by me and he says that I know that I will not get infected because if I was to get infected, I should have long before considering the period of time I have been with you. I don't know where he gets the courage from. I don't know* **(HIV-positive woman)**

#### Seeking Support From Health Workers, Friends, and Relatives

When asked how they eventually remained in a relationship that they had planned to abandon, some couples referred to the post-test counselling support that they received from the health workers (when they went there) while others emphasised the importance of the psychosocial support that they received from their relatives or friends. Individuals that learnt of their HIV-discordant status through the HIVST intervention mentioned that they relied on the support from their relatives or friends to cope with their HIV-discordance. An HIV-negative woman, who learnt about her male partner's HIV-positive status for the first time after she enrolled into the HIVST trial, indicated that when she learnt that her husband was HIV-positive, she called her sister who was in a similar relationship (her sister was HIV-negative while her partner was HIV-positive) who encouraged her to carefully think about the consequences of leaving the relationship, and “*after listening to all this, my sister gave me more courage which I lacked at heart. So I decided to stay and not leave my husband*.” An HIV-negative man with an HIV-positive female partner continued to stay with his HIV-positive female partner after talking to his friend, as illustrated below.

*After learning that she was HIV-positive, I got a friend of mine and I told him about this situation. Just one friend, not a relative but a friend and I told him that you know my wife is HIV-positive but I am negative. He asked me that what are you planning to do and I told him that I had decided to stay with her however I wanted to get advice from you. He told me that looking for a partner is very disturbing and difficult because you could get a partner who will give you hard time. You know women and adultery; women are so much deceived by men who have money. You know what attracts women is wooing them with money…He told me that if your wife understands you, then it is better that you stay with her* **(HIV-negative man)**

However, individuals who already knew their HIV-discordant status indicated that they relied on the post-test counselling support from the health workers to cope with the effects of knowing that they were in an HIV-discordant relationship. Through post-test counselling support, these individuals indicated that they learned of the importance of living harmoniously in an HIV-discordant relationship without “stressing” their partners:

*I received some counselling. What I really got out of this counselling was to ensure that my partner takes medicine. This was something important I got because I leant that when she takes her medicine, the virus will become dormant. And secondly not to stress her and keep reminding her that you are HIV-positive because, just that you have come at a time when she is just recovering from an illness. She had an illness which affected her but before that she was very healthy that no one could even suspect that she is HIV-positive. When she goes to pick her medicine, one could think that she is taking the child for immunisation but she would be getting her medicine. So, what I got from this counselling is that I don't make my partner worried and stressed. This also helped us to stay together because of the fact that you are aware of her status and you know how to support her to stay healthy* **(HIV-negative man)**

When asked what their source of hope for a healthy future is, despite being in an HIV-discordant relationship, HIV-negative participants indicated that the fact their HIV-positive partner is already receiving HIV treatment is a great source of hope. Being on treatment will help their partners to remain healthy and strong and also reduce the risk of HIV transmission to them. Likewise, HIV-positive individuals reported that being on HIV treatment will help them to live a longer life and “*be able to take care of my children*”:

*What gives me hope is being on treatment and to be of importance to my children. What gives me hope is that I am going to be healthy and alive and I will be able to take care of my children when I promptly take my medicine. Also the fact that my partner takes good care of me but even if he doesn't take care of me, I have hope that when I am healthy, I can provide everything I want for myself* **(HIV-positive woman)**

### Current Thoughts and Fears About HIV-Discordance

Although most couples indicated that they were coping well with HIV-discordance, some HIV-negative individuals indicated that they sometimes feared that their partners might one day infect them with HIV, especially if they refused to use a condom. This is a natural feeling of desperation that calls for individuals in HIV-discordant relationships to be provided with on-going post-test counselling.

*Maybe what can come on my mind is sometimes I can sit down and think that since he is sick (i.e., HIV-positive), one day I will also wake up when he has infected me. If he wakes up one morning and [refuses] to use a condom and forces me into sex, won't I risk my life getting infected? Sometimes I get those worries but I try so hard to remain strong* **(HIV-negative woman)**

When asked what their hardest moment has been ever since they learnt about their HIV-discordant status, some HIV-positive individuals expressed fear and scepticism regarding what their partners think about them. In the quotation below, an HIV-positive female partner narrates how she feels that she could be more of a burden to her HIV-negative male partner, something that continues to bother her:

*The hardest thing is sometimes when we are discussing family issues and I get imaginations of what he may be thinking about me in regards to our different status because he does not talk about it. Secondly, sometimes when I am going to get my drugs at the health facility and I do not have transport, I find it hard turning to him for assistance, sometimes I feel like I am a burden and he may complain and get fed up of such a situation* **(HIV-positive woman)**

Although the issue of HIV-discordance has been discussed extensively in health facility settings and through ongoing health education at individual and community levels, misconceptions about HIV-discordance still abound. One participant (an HIV-positive woman) associated HIV infection with the type of blood that she has and believed that when sexual intercourse is fully lubricated, there is no transmission that takes place.

*No, I cannot infect him… with HIV. It all depends on the blood group, there are some blood groups which are resistant and one cannot infect the other with HIV … mine [my blood] rarely infects people* **(HIV-positive woman)**

### Advice to Other Couples in HIV-Discordant Relationships

When asked what advice they would give to other couples with differing HIV status, most participants suggested that such couples should endeavour to avoid getting into arguments with each other (about who brought HIV into the home) but try to support each other to live harmoniously without pointing fingers. There were also suggestions that the HIV-negative partner should support the HIV-positive partner to not only link to HIV care but also to continue to receive and take their HIV medication as expected. HIV-negative partners should continue to protect themselves from the risk of HIV infection and also continue to test for HIV so that if they are found to have acquired HIV, they can be started on HIV treatment as soon as possible. Individuals who are HIV-positive should endeavour to take their treatment as advised rather than resort to regrets. The following quotation illustrates one of the suggestions that can help couples in HIV-discordant relationships to stay together and live harmoniously thereafter.

*I would advise the one who is negative or even request them that you encourage your partner to always go back to the health facility on the return date given and to also make sure that the medicine given is taken as instructed. The other thing is a challenge of getting so worried about the fact that you are positive and your partner is negative and you start thinking that he will even leave me but leaving your partner after going through so much trouble together, I don't see that as right … Lastly, the advice I would give to discordant couples is that the partner who is infected should also take trouble to take his medicine instead of feeling low about themselves saying that after-all I am already infected. The more they continue being reluctant about care, the more their lives will be in danger. Actually, in most cases, people who drop off care get worse and some of them even lose their lives* **(HIV-negative man)**

### Discussion

This study of the coping mechanisms of HIV-discordant couples shows that: (a) most HIV-positive partners thought that their HIV-negative partners would abandon the relationship; (b) couples coped with their HIV-discordant status through: (i) reduced sex frequency/sexual abstinence; (ii) temporary separation from the relationship; (iii) condom use to reduce HIV infection risk to the uninfected partner; and (iv) seeking counselling from health workers or psycho-social support from friends or relatives; (c) some HIV-negative individuals feared that their HIV-positive partners might one day infect them with HIV, especially if they refused to use a condom; and (d) when asked how they would advise other couples in similar situations, participants suggested that such couples should endeavour to avoid getting into arguments with each other (about who brought HIV into the relationship) but try to support each other to live harmoniously without pointing fingers. These findings suggest that, overall, some HIV-discordant couples can eventually outlive the initial fears and frustrations arising from knowing that one of the partners is infected with HIV, especially if they receive post-test counselling from health workers or psychosocial support from people around them. These findings suggest a need to link newly diagnosed HIV-discordant couples to professional post-test counselling services (where these services are available) or to be supported to utilise existing psycho-social support mechanisms to cope with their new HIV-discordant status. Our findings show that newly identified HIV-discordant couples successfully coped with their HIV-discordance through psycho-social support from friends or relatives highlighting the importance of strengthening this component for HIV-discordant couples that learn about their HIV-discordance for the first time through HIV self-testing.

Our findings show that some form of apprehension continues to exist despite the fact that couples appear to be coping well with their HIV-discordant status. Cases of HIV-negative partners fearing that they will eventually be infected with HIV by their HIV-positive partners have been reported in previous studies (7, 21, 22) and could explain the loss of interest in sex that has been reported in some HIV-discordant relationships. Indeed, we found that loss of interest in sex was more common when the HIV-positive partner was male, suggesting that HIV-negative female partners were scared that their HIV-positive male partners could one time infect them with HIV. Male partners in general, and HIV-negative men in particular, seemed not to be bothered with the ongoing HIV infection risk in an HIV-discordant relationship despite encouragement from their female partners to always use a condom during every sexual encounter. Thus, while HIV-negative female partners were always scared of getting infected with HIV from their HIV-positive male partners, some of the HIV-positive male partners did not insist on condom use to protect their HIV-negative female partners from getting infected with HIV. This is the reason that some HIV-negative female partners feared that their HIV-positive male partners will one day infect them with HIV. Likewise, while HIV-positive female partners always encouraged their HIV-negative male partners to use a condom (in order not to infect them with HIV), we documented cases of HIV-negative men that refused to use condoms even if they had them in the house. These findings suggest a need for couple-level interventions that can help HIV-negative and HIV-positive men to realise that the risk of HIV infection to the HIV-negative partner remains apparent despite how long they have stayed together as an HIV-discordant couple. These findings also highlight the need for sensitising HIV-discordant couples about the importance of timely enrolment into HIV care by the HIV-positive partner and timely initiation of PrEP by the HIV-negative partner so as to reduce the risk of HIV infection in such couples.

The fear of being eventually infected with HIV among HIV-negative partners comes amidst findings that show that consistent condom use is not usually possible in long-term sexual relationships (9, 21–24). In the study by Gitonga et al. (21), 23.7% of the couples regarded condom use as “overburdening” and “alien” in the relationship, and cases of partners feeling sexually unsatisfied after using a condom were reported. In another study of initially HIV-negative individuals in HIV-discordant relationships who were eventually infected by their HIV-positive partners, Ngure et al. (25) described cases of HIV-negative men who used condoms inconsistently or not at all (despite the risk of HIV infection from their HIV-positive female partners) while reasoning that condoms were not meant for married people. Some HIV-negative men reported that condom use disrupted their sexual pleasure or that of their partners, despite the apparent risk of HIV infection (25). In another study conducted to document the challenges faced by HIV-negative women in HIV-discordant relationships, HIV-negative women cited the “struggle” that they go through in trying to convince their HIV-positive male partners to use condoms, while noting how difficult it is to negotiate condom use with them (9). Given the difficulty of enforcing condom use in HIV-discordant couples, it might be prudent to encourage HIV-positive individuals to adhere to HIV treatment as much as possible given that adherence to HIV treatment can reduce the risk of HIV infection in HIV-discordant couples to almost zero (6, 26). Our findings also call for a need to promote use of PrEP by HIV-negative members in the relationship to reduce the risk of acquiring HIV infection from their HIV-positive partners (27).

Our study has some limitations and strengths. While we intended to interview 26 individuals from 13 HIV-discordant couples, we were only able to contact 18 individuals. This is likely to have affected the conclusions reported in this paper if the eight individuals that we did not interview had a different set of coping mechanisms than those interviewed, or if they lived in newly identified HIV-discordant couples that still had issues with coping with HIV-discordance. However, we ensured that we interviewed at least one partner in each of the 13 HIV-discordant couples and we hope that this could have helped us to indirectly capture the coping experiences of the partners that we did not interview. This is likely to be true given that most of those interviewed tended to discuss their coping experiences as a couple (i.e., what happened to them and how they coped with it together as a couple) rather than how they coped as individuals. Nevertheless, given that 14 of the 18 individuals interviewed for this study were already aware of their HIV-discordant status at the time of study enrolment, it is likely that by the time of interview, most individuals had already overcome the initial emotions and fears associated with living in an HIV-discordant relationship. Only four individuals indicated that they discovered their HIV-discordant status after they self-tested for HIV. But even then, we did not find any major differences in the way the two sets of couples coped with HIV-discordance, probably because most, if not all, of them had already bypassed the most critical/difficult stage in coping with their HIV-discordant status due to the long duration (18 months) since they last self-tested for HIV. Thus, the coping mechanisms described in this paper should be interpreted with this understanding in mind. We presume that if we conducted the interviews immediately after the couples had just learnt about their HIV-discordant status we could have been able to detect any initial reactions to the HIV-discordant results, especially in couples that did not know that they were HIV-discordant.

The other limitation is that this study was not conducted as a stand-alone study but as part of a large HIVST trial. As a result, the couples that were enrolled into this study were drawn from those that were enrolled into the large HIVST trial to understand how couples identified as HIV-discordant during the trial coped with their HIV-discordant status one-and-a-half-years after the end of the main trial. The number of couples (i.e., 18) to be contacted was determined *a priori* and consisted of at least six couples selected from each of the three study sites where the primary HIVST trial was conducted. As a result, this study did not follow the theoretical sampling procedures (e.g., interview study participants until data saturation is achieved) and the number of interviews conducted was dependent on the availability and willingness of participants drawn from the large HIVST trial to participate in the qualitative study. It is likely that the couples that accepted to participate in this study might have been different from those that refused or were not reached during notification, and this might affect the extent to which these findings represent the coping mechanisms of all HIV-discordant couples enrolled in the main study. However, since qualitative findings tend to be bound in terms of context and time, we believe that the findings shared in this paper reflect the realities that couples go through from the moment they learn about their HIV-discordant status to the time they learn to live harmoniously together as partners in an HIV-discordant relationship.

Despite the above-mentioned limitations, our study has some strengths. For instance, although it was not our intention to study how couples cope with HIV-discordance over a period of one-and-a-half years, we found that the longer that the couples stayed together, the more they devised better strategies for living with their HIV-discordance. This means that if newly diagnosed HIV-discordant couples could be supported to overcome the initial apprehension associated with learning about their HIV-discordant status, there is a great opportunity for these couples to continue to live together for a long time. Such couples can then be supported with HIV prevention interventions such as safe conception to enable them to fulfil their future desires, including fertility desires, without the fear that one or both partners will abandon the relationship. In addition, although only four of the thirteen couples learnt about their HIV-discordance through HIVST, our study's findings provide insights into what goes through the minds of individuals the moment they self-test and learn that they are in an HIV-discordant relationship and how they eventually cope with the situation. However, since these insights are based only on a few couples, future research is still needed to fully appreciate how couples cope with HIV-discordant results after HIVST.

## Conclusion

Our study shows that although HIV-discordant results initially cause tension in the relationship, this tension eases with time. Couples coped with their HIV-discordant status through reducing the frequency of sex or abstaining from sex altogether; temporarily leaving the relationship; using condoms to reduce the risk of HIV infection to the uninfected partner, and seeking post-test counselling from health workers or psychosocial support from friends or relatives. These findings suggest a need for programs that offer HIVST services to create avenues for linking newly identified HIV-discordant couples to post-test counselling support (where possible) or, where this is not possible, for them to be supported to seek psycho-social support from their relatives or friends. It is important to note that a few couples continued to experience tensions resulting from the fear that the HIV-negative partner would eventually be infected with HIV by the HIV-positive partner and some couples reported total failure in using condoms despite the ongoing risk of HIV infection. These findings suggest a need for continued follow-up support to couples in HIV-discordant relationships to help them to cope with their HIV-discordant status. Supporting HIV-positive partners to enrol into HIV care immediately after HIV diagnosis and the HIV-negative partners to initiate PrEP could be one way of helping HIV-discordant couples to continue to cope with their HIV-discordant status.

## Data Availability Statement

The datasets presented in this article are not readily available because this study collected qualitative data that may not be completely de-identified. For that reason, we are unable to share the data. Requests to access the datasets should be directed to jmatovu@musph.ac.ug.

## Ethics Statement

The primary study, which involved human participants, was reviewed and approved by the Higher Degrees Research and Ethics Committee of Makerere University School of Public Health, Kampala, Uganda. The study protocol was also registered with the Uganda National Council for Science and Technology in line with the national research regulations. Participants provided written informed consent to participate in this study.

## Author Contributions

JKBM, RK, JEK, and RKW conceived the study. JKBM and RK implemented, supervised the study, and performed the data analysis. JKBM drafted the manuscript. JKBM, RK, JEK, AMM, CV-D, SCM, WM, and RKW critically revised the manuscript for substantial intellectual content. All authors read and approved the final manuscript.

## Conflict of Interest

The authors declare that the research was conducted in the absence of any commercial or financial relationships that could be construed as a potential conflict of interest.

## Publisher's Note

All claims expressed in this article are solely those of the authors and do not necessarily represent those of their affiliated organizations, or those of the publisher, the editors and the reviewers. Any product that may be evaluated in this article, or claim that may be made by its manufacturer, is not guaranteed or endorsed by the publisher.
